# Dynamics of neuroinflammation in the macrosphere model of arterio-arterial embolic focal ischemia: an approximation to human stroke patterns

**DOI:** 10.1186/2040-7378-2-22

**Published:** 2010-12-20

**Authors:** Maureen Walberer, Maria A Rueger, Marie-Lune Simard, Beata Emig, Sebastian Jander, Gereon R Fink, Michael Schroeter

**Affiliations:** 1 Department of Neurology, University Hospital, Cologne, Germany; 2 Max-Planck-Institute for Neurological Research, Cologne, Germany; 3 Institute of Neuroscience and Medicine (INM-3), Cognitive Neurology Section, Research Centre Juelich, Germany; 4 Department of Neurology, Heinrich-Heine-University, Düsseldorf, Germany

## Abstract

**Background:**

Neuroinflammation evolves as a multi-facetted response to focal cerebral ischemia. It involves activation of resident glia cell populations, recruitment of blood-derived leucocytes as well as humoral responses. Among these processes, phagocyte accumulation has been suggested to be a surrogate marker of neuroinflammation. We previously assessed phagocyte accumulation in human stroke by MRI. We hypothesize that phagocyte accumulation in the macrosphere model may resemble the temporal and spatial patterns observed in human stroke.

**Methods:**

In a rat model of permanent focal ischemia by embolisation of TiO_2_-spheres we assessed key features of post-ischemic neuroinflammation by the means of histology, immunocytochemistry of glial activation and influx of hematogeneous cells, and quantitative PCR of TNF-α, IL-1, IL-18, and iNOS mRNA.

**Results:**

In the boundary zone of the infarct, a transition of ramified microglia into ameboid phagocytic microglia was accompanied by an up-regulation of MHC class II on the cells after 3 days. By day 7, a hypercellular infiltrate consisting of activated microglia and phagocytic cells formed a thick rim around the ischemic infarct core. Interestingly, in the ischemic core microglia could only be observed at day 7. TNF-α was induced rapidly within hours, IL-1β and iNOS peaked within days, and IL-18 later at around 1 week after ischemia.

**Conclusions:**

The macrosphere model closely resembles the characteristical dynamics of postischemic inflammation previously observed in human stroke. We therefore suggest that the macrosphere model is highly appropriate for studying the pathophysiology of stroke in a translational approach from rodent to human.

## Background

Inflammation plays an important role in the cascade of events following cerebral ischemia that may impact on the extent of tissue damage, infarct demarcation, tissue repair und functional recovery, and may hence act as a key target for therapeutic intervention [1,2].

Animal research has characterized postischemic inflammation as a multi-facetted response involving activation of resident glia cells and recruitment of blood-derived leucocytes as well as cascades of humoral responses [2-4]. In the classical transient middle cerebral artery occlusion (tMCAO) model, hematogeneous cells including polymorphonuclear neutrophils (PMN) and macrophages rapidly infiltrate the ischemic region [5-7].

Translating rodent research into the situation of human stroke, substantial progress has been made in visualizing aspects of postischemic inflammation in man. Starting with the first *in vivo *visualization of peripheral benzodiazepine receptor-expressing inflammatory cells using Positron Emission Tomography (PET) and the radiotracer [^11^C]PK11195 [8], postischemic inflammation has been repeatedly characterized by PET [8-11], magnetic resonance imaging (MRI) [12], and cell specific contrast agents detected by MRI [13-15]. Early histopathological descriptions suggest a significant impact of permanent versus transient ischemia on the dynamics of inflammation [6]. Additionally, in the classical tMCAO model, tissue damage and glia activation evolve in complex spatial and temporal dynamics that make it difficult to interpret the results and to translate them into the human situation [16,17]. Different dynamics of MRI signatures in the tMCAO model and human stroke further complicate a translational approach [15]. Accordingly, we searched for an experimental stroke model with particular regard to the dynamics of postischemic inflammation that resembles the human situation.

In the rat macrosphere model, permanent focal ischemia is induced by intra-arterial embolization of a defined number of TiO_2 _spheres into the middle cerebral artery (MCA). With respect to parameters such as infarct development over time, final lesion size and clinical outcome, this model is comparable to the established pMCAO (permanent MCAO) model using an intraluminal thread [18,19]. However, in contrast to the permanent suture model, hypothalamic injury followed by pathological hyperthermia is avoided in the macrosphere model [20,21]. Moreover, macrosphere injection mimics arterio-arterial embolism of arteriosclerotic plaque material as the leading etiology of human stroke, while the thread occlusion model rather simulates a thromboembolic event with subsequent thrombolysis and large-vessel reperfusion [22]. Importantly, neuroinflammation has not been described in an arterio-arterial embolic stroke model to date.

Hence, we investigated key features and dynamics of postischemic inflammation in the macrosphere model and compared our findings to the human situation as revealed by previous PET and MRI Studies.

## Methods

### Animals and Surgery

**Figure 1:**
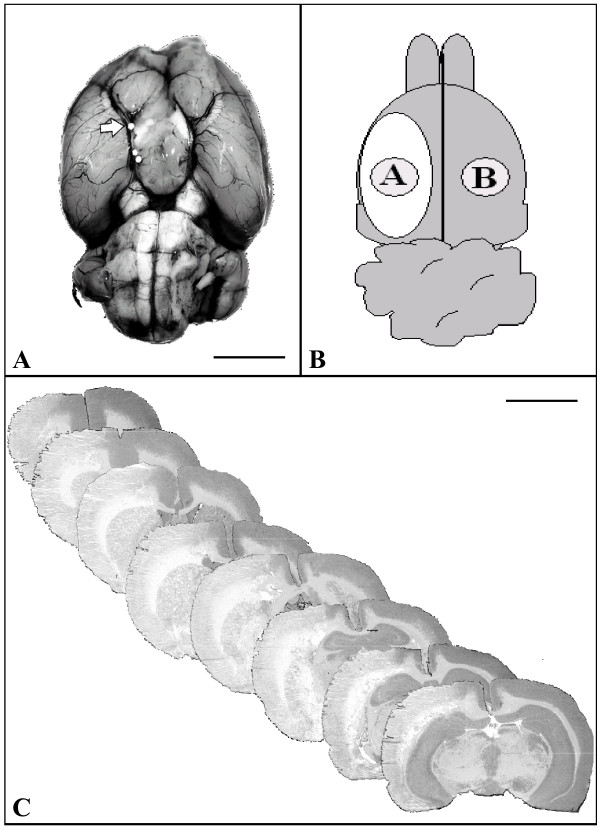
**Characterization of the macrosphere stroke model**. (A) Basal view of the rat brain displaying the cerebral arteries following macrosphere-injection into the MCA. One macrosphere is located in the origin of the MCA and the distal ICA (arrow). Bar: 5000 μm. (B) Schematic drawing of the rat brain (dorsal view) to localize tissue samples collected from the ischemic lesion [A] and contralateral cortex [B]. (C) Representative H&E staining of coronal brain sections demonstrating the extent of the ischemic lesion 7 days after ischemia induction. Objective 1×; bar: 5000 μm.

All animal procedures were in accordance with the German Laws for Animal Protection and were approved by the local animal care committee and local governmental authorities. Male Wistar rats (n = 33) weighing 270-340 g were anesthetized with 5% isoflurane and maintained with 2.5% isoflurane in 65%/35% nitrous oxide/oxygen. Throughout the surgical procedure body temperature was maintained at 37.0°C with a thermostatically controlled heating pad. Ischemia was produced by intra-arterial injection of 4 TiO_2 _spheres into MCA as described elsewhere [19]. Briefly, after exposure of the left common carotid artery (CCA), internal carotid artery (ICA) and external carotid artery (ECA), the ECA and the pterygopalatine branch of the ICA were ligated. PE-50 tubing was filled with saline and four TiO_2 _macrospheres (Ø 0.315-0.355 mm; BRACE, Alzenau, Germany). The macrospheres were advanced via ICA into the MCA by a slow injection of approximately 0.2 ml saline (Figure 1A). In sham-operated animals, the same operation procedure was used without injection of macrospheres (n = 4). Following operation, the animals were transferred to their cages after they had fully recovered from anaesthesia. During the experiment all animals received an intensified care with subcutaneous saline injections (5 ml 0.9%NaCl/day) and moistening of food pellets. Animals in apparent clinical distress were sacrificed, excluded from the study and replaced.

### Histology and Immunocytochemistry

Groups of n = 3 animals each were allowed to survive for 24 hours, 3 days, and 7 days after MCA embolisation with TiO_2 _spheres before they were decapitated under deep anaesthesia with isoflurane. The brains were rapidly removed, frozen in 2-methylbutane, and stored at -80°C prior to further histological and immunocytochemical processing. Adjacent serial coronal brain sections were cut at 500 μm intervals (slice thickness 10 μm) and stained with hematoxylin and eosin (H&E) according to standard protocols. Anti-NeuN (clone A60, dilution 1:1000, Millipore, Billerica, USA, cat-# MAB377) was performed to recognize the DNA-binding between neuron and the specific protein NeuN, which is presented in most CNS/PNS neuronal cell types. For the identification of astrocytes, we used mAb against Glial Fibrillary Acidic Protein (GFAP) (clone GFAP, dilution 1:1000, Millipore, Billerica, USA, cat-# MAB360) and Vimentin (VIM) (dilution 1:2000, Millipore, Billerica, USA, cat-# MAB3400). The mAb against the complement receptor 3/CD11b identified microglia/macrophages (clone OX42, dilution 1:1000, AbD Serotec, Oxford, UK, cat-# MCA275R). Microglia activation was assessed by staining for MHC class II (clone Ox6, dilution 1:400, AbD Serotec, Oxford, UK, cat-#MCA46G). Phagocytic cells were identified with mAb ED1 (clone ED1, dilution 1:1000, AbD Serotec, Oxford, UK, cat-# MCA341). A double staining with anti-Iba1 (dilution 1:1000, Wako, Neuss, Germany, cat-# 019-19741) and anti-NeuN was performed additionally. Iba1 is up-regulated upon activation of microglia allowing the discrimination between resting and activated microglia. For visualization, the ABC Elite kit (Vector Laboratories, Burlingame, CA, USA) with diaminobenzidine (Sigma, Munich, Germany) or Vector SG substrate kit for peroxidase (Vector laboratories, Burlingame, CA, USA.) for anti-NeuN staining as the final reaction product was used.

To quantify phagocytic cells at day 7, the area of ED1-positive cells was measured by the analysis software Image J (1.40G, National Institutes of Health, USA) and related to the ipsilateral hemisphere.

### Quantitative real-time polymerase chain reaction (PCR)

For PCR analysis, another group of animals were sacrificed 4 hours (n = 5), 24 hours (n = 5), 3 days (n = 5), and 7 days (n = 5) after ischemia induction. In addition, 4 sham-operated animals without infarct served as controls. For RNA isolation, a sample of the cortex within the infarct core (sample A) and contralateral hemisphere (sample B) were prepared along with non-ischemic cortex from control brains (Figure 1B). Approximately 30 to 50 mg tissue per tissue sample (wet weight) were obtained. Total RNA was prepared using the Trizol reagent (Gibco BRL, Gaithersburg, MD) and quantified spectrophotometrically. One microgramm RNA was reversely transcribed using oligo (dT)_19 _(G/A/C) primers and Superscript II reverse transcriptase (Gibco-BRL, Gaithersburg, MD) according to manufacturer's protocol. Quantitative assessment of TNF-α, IL-1β, IL-18 and iNOS gene expression levels was performed using a 7900HT Fast Real-Time PCR System (Applied Biosystems, Weiterstadt, Germany) and the Sybr Green Universal Master Mix (Applied Biosystems) and gene-specific primer pairs as described previously [23,24]. In all PCR analyses, glyceraldehyde 3-phosphate dehydrogenase (GAPDH) [24] was used as the reference gene since it exhibited constant expression levels under all tested conditions. Relative gene expression levels were determined according to the manufacturer's ΔΔ*C*_t _method. For quantification of PCR data, mRNA levels of the ipsilateral hemisphere were compared to levels of the contralateral hemisphere as well as to values of control animals.

### Statistical analysis

The statistical analysis was performed with SigmaPlot 11 (Systat Software Inc, California, USA). Data are presented as mean ± SD. For the analysis of PCR results, two-way analysis of variance (ANOVA) with a post hoc test of Holm-Sidak was used. Statistical significance was set at the less than 5% level (p < 0.05).

## Results

### Characterization of the macrosphere model

Four TiO_2 _spheres were injected into the ICA after ligation of the pterygopalatine artery, leading to a permanent occlusion within the ICA/MCA vessel arborisation. In all animals, one or more macrospheres directly blocked the proximal MCA (Figure 1A). H&E staining verified the infarct localization in the MCA territory of all animals (Figure 1C).

All animals showed circling behavior 24 hours after infarct induction but recovered incompletely till 72 hours. Animals in clinical distress were sacrificed, excluded from the study and replaced (n = 4).

### Infarct demarcation

**Figure 2:**
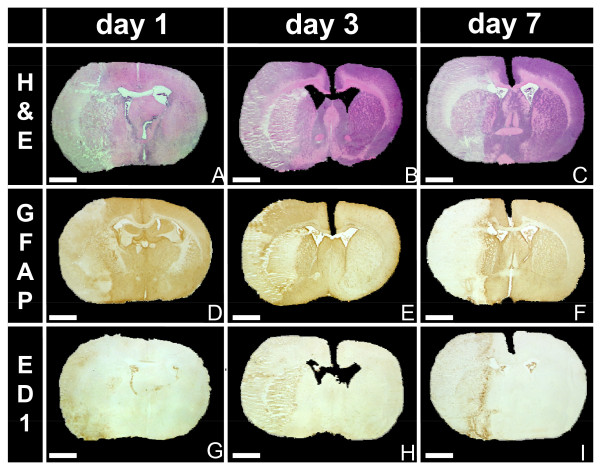
**Infarct demarcation and cellular response**. H&E (A-C), GFAP (D-F) and ED1 (G-I) staining 24 h, 3 d, and 7 d after infarct induction. The infarct is increasingly demarcated over time by reactive astrocytes that accumulate in the vital tissue bordering the infarct. Scars circumscribe the infarcted tissue but GFAP-positive cells do not enter the infarct zone up to day 7. Phagocytic cells including transformed microglia and hematogeneous macrophages accumulate at the infarct border in the necrotic tissue. Objective: 1×; bar: 200 μm.

One day after induction of stroke, the infarction is already well demarcated from vital tissue (Figure 2A, D, G). H&E staining showed eosinophilic coagulation necrosis in the ischemic territory (Figure 2A) and loss of GFAP staining indicated cell loss and edema within the infarct core (Figure 2D). The infarct border is even more clear-cut 3 days after macrosphere injection (Figure 2B, E, H) and the surrounding tissue appears to be slightly hypercellular. Around day 7, a sharp infarct demarcation with a surrounding hypercellular infiltrate containing reactive astrocytes (revealed by GFAP) and phagocytic cells containing transformed microglia and hematogeneous macrophages (identified by ED1) developed (Figure 2C, F, I). The dynamics of infarct demarcation and astrogliosis did not differ between subcortical and cortical regions at the observation time-points, giving further evidence to the notion that the infarcts developed promptly and in a synchronous way in basal ganglia and cortical areas.

The mean ischemic lesion volume of all animals was 161 mm^3 ^± 90.

The contralateral hemisphere did not show any changes in immunoreactivity at any time point (Figure 2).

### Cellular inflammatory response

**Figure 3:**
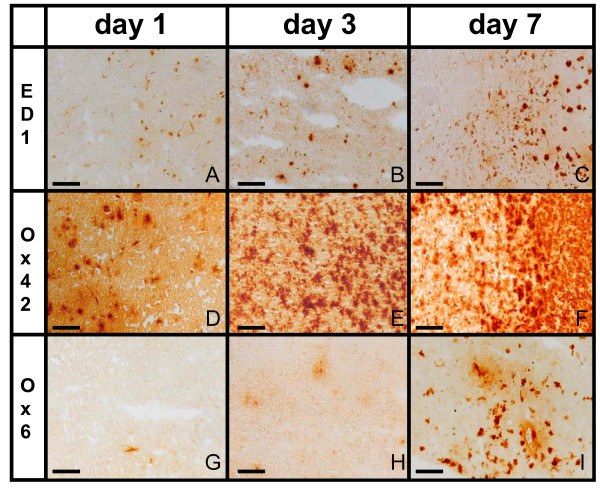
**Cellular inflammatory response at the infarct border**. Phagocytic cells (ED1, A-C), microglia (Ox42, D-F) and activated microglia (Ox6, G-I) are responding to focal cerebral ischemia. Over the course of one week after induction of ischemia, those cells accumulated at the infarct border. Objective: 10×; bar: 100 μm.

In the infarct core morphological signs of microglial activation could be shown as early as 24 h after induction of focal ischemia by more intense Ox42-staining. Some amoeboid microglia with rounded cell bodies were also present (Figure 3A, D, G). By day 3, microglia cells started to form a hypercellular rim around the infarct core, exhibiting signs of activation by ED1-staining as an indication for phagocytosis. MHC class II-positive cells with stellate morphology were more numerous and extended further into vital tissue compared to ED1-positive cells. (Figure 3B, E, H). At day 7, a dense cellular wall of microglial cells surrounded the ischemic lesion (Figure 3C, F, I). In the core of the lesion, some - most likely avital - cells exhibited a granular Ox42 immunosignal. There was abundant expression of ED1 and MHC class II-positive cells constituting a rim around the infarct border, with more scattered cells positive for both markers at the infarct core. At day 7, the area of ED1-positive cells (54 mm^3 ^± 26) extended over 9 to 20% of the affected hemisphere.

The contralateral hemisphere did not show changes in immunoreactivity at any time point.

### Cellular interactions in the infarct demarcation zone

**Figure 4:**
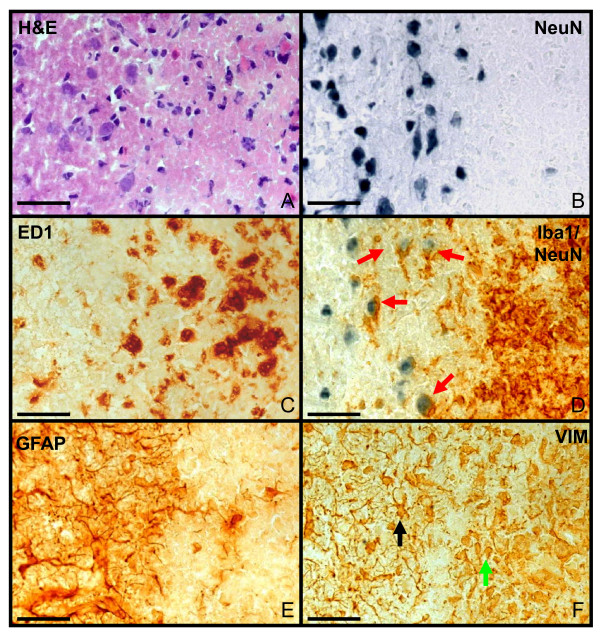
**Cellular interaction in the infarct demarcation zone**. Infarct demarcation zone at day 7 after infarct induction (left: healthy tissue; right: infarct zone) with different stainings: H&E (A), NeuN (B), ED1 (C), anti NeuN antibody and anti-Iba1 antibody (D), GFAP (E) and VIM (F). The infarct zone was clearly demarcated (A-F) Eosinophilic coagulation (A) and neuronal degeneration (B) occurred at the infarct. ED1+ phagocytes (C) and activated microglia (D, brown) are visible within the infarct. Interactions between neurons and activated microglia occurred (D, red arrows). GFAP-positive astrocytes (E) demarcate the lesion forming an astrocytic scar in the vital tissue adjacent to the infarct border. Vimentin immunoreactivity (F) depicts proliferating astrocytes within the scar (black arrow) as well as other cell types within the infarct (green arrow). Objective: 40×; bar: 50 μm.

7 days after MCAO, eosinophilic coagulation necrosis (Figure 4A) and neuronal degeneration (Figure 4B) could be observed in the infarct core. ED1+ phagocytes (Figure 4C) and activated microglia (Figure 4D, **brown**) accumulated at the infarct border and interacted with neurons (Figure 4D, **red arrows**). Astrocytic activation (protoplasmic astrocytes) was clearly visible at the border of the infarct, but not in the infarct core. A demarcation of a scar-like formation could be observed at the periphery of the infarct core (Figure 4E, F) and vimentin immunoreactivity depicted immature astrocytes indicating proliferation of astrocytes.

### Cytokine response

**Figure 5:**
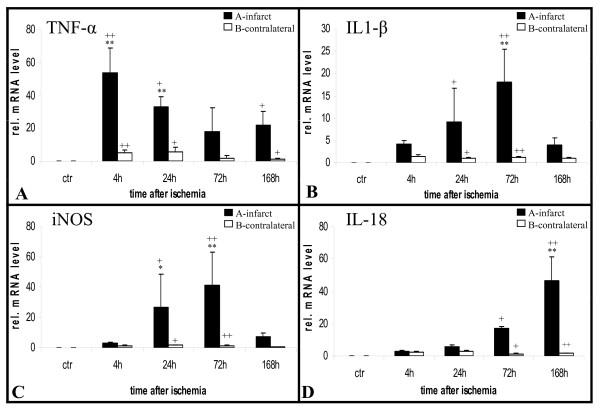
**Cytokine response**. Quantitative real-time polymerase chain reaction (PCR) analyses of TNF-α (A), IL-1β (B), iNOS (C) and IL-18 (D) mRNA levels shows upregulation of all cytokines within specific time windows. A rapid strong induction of TNF-α peaking at 4 h was accompanied by a lower response of IL-1β and iNOS with a maximum at 72 h. IL-18 was induced very late around day 7. At each time point after onset of infarct, n = 5 animals were studied, whereas 4 animals served as control. Bars represent mean ± SD; **p < 0.01; *p < 0.05 (compared to control animals); ^++^p < 0.01; ^+^p < 0.05 (infarcted hemisphere compared to contralateral hemisphere).

The proinflammatory cytokines TNF-α, IL-1β, iNOS and IL-18 were quantified in the infarct core and homotopic contralateral regions over time (Figure 1B). Permanent MCAO induced the early upregulation of TNF-α mRNA within the first 4 hours after ischemia. TNF-α stayed upregulated at significant levels for the entire time period studied (Figure 5A). IL-1β and iNOS were upregulated at a slower rate and peaked 72 hours after onset of ischemia (Figure 5B, C). IL-18 expression was detectable at low levels within the first days and peaked at 7 days after stroke (Figure 5D).

## Discussion

In the macrosphere model, the intra-arterial injection of 4 TiO_2 _spheres leads to permanent occlusion of the MCA resulting in an ischemic lesion of the MCA-territory [20]. The interindividual variability of infarct volume caused by different occlusion sites of the macrospheres is similar to other embolic stroke models. This technique mimics arterio-arterial embolism of "hard" atherosclerotic plaque material, the most frequent cause of stroke in humans [22,25]. Moreover, in contrast to the well-established suture model of permanent ischemia, the macrosphere model avoids hypothalamic injury and subsequently hyperthermia [18,20,21], while hypothalamic infarction is extremely rare in patients. Hyperthermia may be a confounding factor in therapeutic studies and may influence CNS inflammatory responses as well. In those regards, the macrosphere model offers clear advantages compared to the commonly used transient and permanent suture models, and has therefore been suggested to represent a clinically relevant model to study the pathophysiology of stroke.

In a proof-of-principles study we previously visualized macrophage infiltration in human stroke by a superparamagnetic iron oxide contrast agent (USPIO) [14]. At 3-4 days after stroke onset, we detected subtle contrast enhancement in only a minority of patients. However, 6-8 days after onset of ischemia, we consistently found signs of macrophage infiltration in subcortical as well as cortical areas. Macrophage infiltration heterogeneously affected subregions of the infarct with significant inter-individual differences. This is in accordance with the dynamics of cellular responses after permanent focal ischemia in primates reported earlier [26]. Going back from "bedside to bench", two essential features of cellular responses in the macrosphere model are reminiscent of the human situation: (i) the slowly evolving macrophage infiltration at the end of the first post-infarct week, and (ii) the heterogeneous distribution of the macrophage infiltrate throughout the infarct.

In rodent models of tMCAO, the temporal evolution of tissue damage has also been shown to be heterogeneously, extending in distinct patterns on a subcortical to cortical axis, which renders inflammatory responses complex and difficult to assess [16,17]. Reperfusion accelerates inflammation, facilitating the rapid infiltration of hematogeneous cells, to an early and diffuse accumulation of PMNs throughout the infarct [5-7,27].

In contrast, rodent models of permanent MCAO show rapid signs of tissue damage evolving in the infarct core and succumbing to necrosis, while more peripheral areas display a greater variability in the severity of ischemia. After pMCAO, peripheral areas are usually associated with incomplete necrosis, while subcortical regions show only mild ischemic neuronal damage without loss of neurons [28]. In a rat model of pMCAO, direct surgical occlusion of the MCA led to an accumulation of PMN leukocytes with a maximum at 48-72 hours [16], which is just as late as the PMN infiltration seen after human stroke [7]. In a monkey model of pMCAO, PMNs within the reactive zone could be observed in modest numbers at 18 hours, and increased to 72 hours, while seven-day old lesion showed PMNs in the central infarcted zone only [16]. By comparing permanent and transient MCAO in spontaneously hypertensive rats, the infiltration of neutrophils was moderate in pMCAO. Furthermore, the activity of the neutrophil marker myeloperoxidase was 2 to 3-fold increased, depending of the reperfusion time in tMCAO compared to pMCAO within the first day [27]. Those previous studies suggest that pMCAO models reflect the dynamics of human postischemic inflammation much better than tMCAO models do.

In this study, the extent of ischemic damage obtained by macrosphere embolization corresponded to previous experiments and was comparable to other pMCAO models [18-21]. In contrast, our pMCAO model was different from the previously described photochemically induced focal ischemia model that produces only a pure neocortical infarction [29,30]. While the infarcted area underwent necrosis, the surrounding tissue displayed an increasing astrocyte reactivity over time as revealed by GFAP-staining, a phenomenon that was similar to photothrombosis [30,31]. With respect to the cellular inflammatory response, we observed a transition of ramified into ameboid phagocytic microglia in the boundary zone of the infarct, with consecutive infarct demarcation as late as three days after the induction of ischemia, corresponding to the situation after photothrombosis [29] as well as to that after human stroke [4]. Stoll et al. observed the accumulation of phagocytes in the border zone of human infarcts after 5 days [4]. This combined microglia/macrophage response was accompanied by an up-regulation of MHC class II molecules. In the ischemic core, morphological signs of microglia activation could be detected by day 7, but not at day 1, similar to the photothrombosis model [17,30,32,33]. In human PET studies using the peripheral benzodiazepine ligand [^11^C]PK11195 as a surrogate marker for inflammation to investigate the temporo-spatial profile of microglia-activation and macrophage-invasion [34-36] and MRI studies using ultrasmall supermagnetic iron oxide (USPIO) to study the invasion of blood-borne macrophages into human brain [12,37], microglia activation in human stroke have been detected to start as late as 3 days after onset of ischemia and reached its maximum within one week [12,34-37]. Interestingly, in the macrosphere model activated microglia and macrophages established a particularly dense cellular wall around the infarct core after 7 days, whereas reactive astrocytes had formed a ring already by day 3, similar to the situation observed in the photothrombosis model [29,31]. The time-course of cytokine release in the macrosphere model differed slightly from the situation in the photothrombosis model. While we found a rapid induction of TNF-α similar to photothrombosis, a much later upregulation of IL-1β and iNOS occurred peaking at day 3. TNF-α and IL-1β are synthesized by PMNs [38] and therefore PMN accumulation may contribute to TNF-α and IL-1β production-besides resident microglia as a principle source-peaking at 72 hours. The induction of IL-18 occurred even later and increased up to day 7. This late induction of IL-18 is in good accordance with the situation previously observed in the photothrombosis model, where the maximum upregulation was found at day 14, a time point not included in our present study [23].

By comparing macrosphere model, human stroke and tMCAO, key features of post-ischemic neuroinflammation, e.g. microglia activation, macrophages infiltration throughout the infarct and phagocytic accumulation, showed a similar temporal appearance in the macrosphere model in rats and human stroke, whereas tMCAO in rats leads to a rapid development of inflammation (Table 1).

**Table 1:** Cytokine response

	Macrosphere model	Human stroke	tMCAO
**Activation of microglia (maximum)**	day 3 (day 7)	day 3 (day 7)^37^	6 h (day 3)^17^
**Transformation from ramified to ameboid microglia in the infarct border**	from day 3 on	days?	22 h^17^
**Infiltration of macrophages/microglia in the infarct core**	day 7	day 6- day 8^14^	6 h^17^
**Spatial patterns of macrophages/microglia accumulation**	"wall-like" accumulation in the outer infarct, sparing the infarct core	"wall-like" accumulation in the outer infarct, sparing the infarct core^14^	heterogeneous, extending in distinct patterns on a subcortical to cortical axis^17^
**Accumulation of phagozytes**	day 7	day 5-8^4^	day 3^17^

Time-points of various signs of inflammation after focal cerebral ischemia in macrosphere model, human stroke and tMCAO

## Conclusions

The macrosphere model as a model of focal cerebral ischemia resembles closely the dynamics of human postischemic inflammation, imitating particularly the slow time course of human neuroinflammation. Therefore we suggest that the macrosphere model mimics the clinical situation of human stroke better than the commonly used model of transient middle cerebral artery occlusion. Accordingly the rodent macrosphere model is regarded most relevant for studying the pathophysiology of stroke and possesses high clinical relevance.

## Competing interests

The authors declare that they have no competing interests.

## Authors' contributions

MW made the conception and design of the study, carried out animals' surgery, participated in the acquisition of histological and PCR-data, performed statistical analysis of data and drafted the manuscript. MAD participated in the acquisition and interpretation of histological data and helped to draft the manuscript. MLS Simard participated in the acquisition of histological data. SJ participated in analysis and interpretation of PCR-data. GRF revised the manuscript and has given final approval of the version to be published. MS conceived the study, helped with the interpretation of histological data and revised the manuscript critically for important intellectual content. All authors read and approved the final manuscript.
